# Mechanism for enhanced 5-aminolevulinic acid fluorescence in isocitrate dehydrogenase 1 mutant malignant gliomas

**DOI:** 10.18632/oncotarget.4060

**Published:** 2015-05-10

**Authors:** Ja Eun Kim, Hye Rim Cho, Wen Jun Xu, Ji Young Kim, Sung Kwon Kim, Seung-Ki Kim, Sung-Hye Park, Hyeonjin Kim, Se-Hoon Lee, Seung Hong Choi, Sunghyouk Park, Chul-Kee Park

**Affiliations:** ^1^ Department of Neurosurgery, Seoul National University College of Medicine, Seoul National University Hospital, Jongno-gu, Seoul, Korea; ^2^ Department of Radiology, Seoul National University College of Medicine, Seoul National University Hospital, Jongno-gu, Seoul, Korea; ^3^ College of Pharmacy, Natural Product Research Institute, Seoul National University, Sillim-dong, Gwanak-gu, Seoul, Korea; ^4^ Department of Neurosurgery, Gyeongsang National University School of Medicine, Gyeongsang National University Hospital, Jinju, Korea; ^5^ Division of Pediatric Neurosurgery, Seoul National University Children's Hospital, Jongno-gu, Seoul, Korea; ^6^ Department of Pathology, Seoul National University College of Medicine, Seoul National University Hospital, Jongno-gu, Seoul, Korea; ^7^ Division of Hematology-Oncology, Department of Medicine, Samsung Medical Center, Sungkyunkwan University School of Medicine, Seoul, Korea

**Keywords:** brain tumors, oncology, 5-ALA, fluorescence, IDH1, glioma, NADPH

## Abstract

Fluorescence-guided surgery using 5-aminolevulinic acid (5-ALA) has become the main treatment modality in malignant gliomas. However unlike glioblastomas, there are inconsistent result about fluorescence status in WHO grade III gliomas. Here, we show that mutational status of *IDH1* is linked to 5-ALA fluorescence. Using genetically engineered malignant glioma cells harboring wild type (U87MG-IDH1^*WT*^) or mutant (U87MG-IDH1^*R132H*^) *IDH1*, we demonstrated a lag in 5-ALA metabolism and accumulation of protoporphyrin IX (PpIX) in U87MG-IDH1^*R132H*^ cells. Next, we used liquid chromatography–mass spectrometry (LC-MS) to screen for tricarboxylic acid (TCA) cycle-related metabolite changes caused by 5-ALA exposure. We observed low baseline levels of NADPH, an essential cofactor for the rate-limiting step of heme degradation, in U87MG-IDH1^*R132H*^ cells. High levels of NADPH are required to metabolize excessive 5-ALA, giving a plausible reason for the temporarily enhanced 5-ALA fluorescence in mutant *IDH1* cells. This hypothesis was supported by the results of metabolic screening in human malignant glioma samples. In conclusion, we have discovered a relationship between enhanced 5-ALA fluorescence and *IDH1* mutations in WHO grade III gliomas. Low levels of NADPH in tumors with mutated *IDH1* is responsible for the enhanced fluorescence.

## INTRODUCTION

Among recent advances in malignant glioma surgery, fluorescence-guided surgery is becoming popular due to its ability to improve the extent of resection while preserving patient functional status [1, 2]. 5-aminolevulinic acid (5-ALA) highlights otherwise indistinguishable malignant areas with a temporarily red fluorescence under an appropriate blue light source with a specific wavelength [3]. 5-ALA itself is a non-fluorescent endogenous compound that can be metabolized intracellularly into protoporphyrinogen IX (PpIX), a fluorescent intermediate product of the heme synthesis pathway [4]. Although the exact mechanism responsible has yet to be verified, exogenous administration of 5-ALA leads to selective accumulation of PpIX in malignant gliomas but not in low-grade gliomas or normal brain tissue [5]. The 5-ALA-induced fluorescence rate is lower in WHO grade III gliomas (83% in anaplastic oligodendroglioma/oligoastrocytomas, and 22% in anaplastic astrocytomas) than grade IV gliomas (96% in glioblastomas) [6]. The reason why grade III gliomas have a lower 5-ALA fluorescence rate than grade IV gliomas is unknown.

The identification of isocitrate dehydrogenase 1 *(IDH1)* mutations in glioma is one of the major discoveries mediating metabolomics and oncogenesis [7]. The majority of *IDH1* mutations occur at the active site arginine 132, the most common substitution being histidine (R132H) [7-10]. The role of *IDH1* is to catalyze the oxidative decarboxylation of isocitrate into α-ketoglutarate (α-KG), simultaneously converting NADP(+) to NADPH in the cytoplasm and peroxisomes [11, 12]. *IDH1* mutations in glioma cells diminish the canonical enzymatic activity of IDH1 while conferring a neomorphic enzymatic activity: the production of R-2-hydoxyglutarate (2-HG) via the consumption of NADPH [13-16]. The *IDH1* mutation is observed in 6% of WHO grade IV glioblastomas and 55% of grade III gliomas [17].

Endogenously, 5-ALA is produced from succinyl-CoA, which is converted from α-KG [18]. Based on the idea that *IDH1* mutations can alter the 5-ALA metabolic pathway and stimulate exogenous 5-ALA fluorescence, we confirmed an association between the R132H *IDH1* mutation and enhanced 5-ALA fluorescence in WHO grade III gliomas, then investigated metabolic aspects of the underlying mechanism. Although the *IDH1* mutation is not the core mechanism of 5-ALA fluorescence considering the discrepancy between 5-ALA fluorescence and *IDH1* mutation prevalence in grade II or IV gliomas, we showed that the *IDH1* mutation acts as a sensitizer when the cell is under the condition of altered 5-ALA metabolism.

## RESULTS

### Clinical evidence of an association between IDH1 mutations and 5-ALA fluorescence

Among 35 patients with WHO grade III gliomas who were operated upon using 5-ALA fluorescence-guided surgery, *IDH1* mutations were observed in 24 and intraoperative fluorescence was observed in tumor tissues of 19 patients (Table 1). There was a significant association between *IDH1* mutations and 5-ALA fluorescence in tumor tissue (*p* = 0.03). The distribution of patient data and associations between variables are shown in a mosaic plot (Figure 2).

**Table 1:** Association between intraoperative 5-ALA fluorescence and IDH1 mutations in WHO grade III glioma patients

	Intraoperative fluorescence		Total
	Yes	No	
Mutated *IDH1*	16	8	24
Wild type *IDH1*	3	8	11
Total	19	16	35

*p* = 0.03

### 5-ALA metabolism lags in IDH1 mutant malignant glioma cell lines

**Figure 1:**
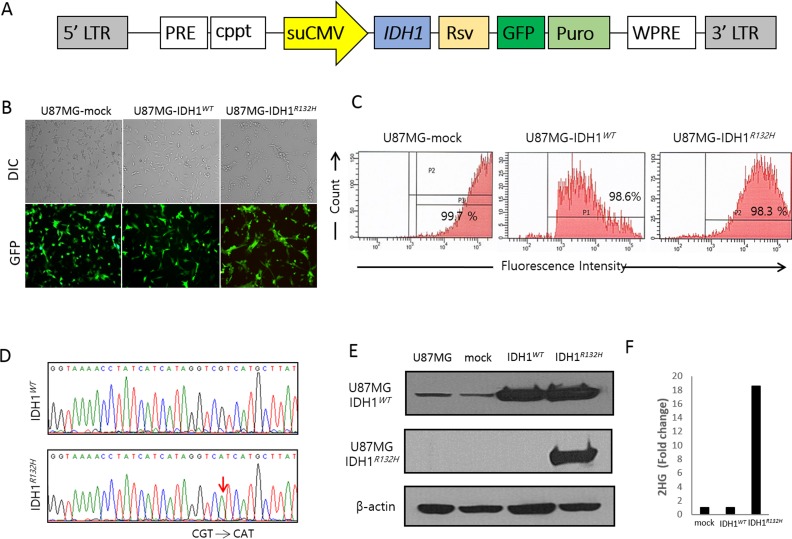
Establishment of wild type and mutant *IDH1* cell lines using U87MG cells and a lentiviral vector **A.** Schematic drawing of the lentiviral *IDH1* gene construct. **B.** Transduction efficacy of vector containing the *IDH1* gene construct, evaluated by GFP. **C.** FACS results showing high purity of GFP-positive cells. **D.** Direct sequencing analysis confirming expected sequences in U87MG-IDH1*^WT^* and U87MG-IDH1*^R132H^* cells. **E.** Western blot results showing expression of IDH1*^WT^* and IDH1*^R132H^* from U87MG-IDH1*^WT^* and U87MG-IDH1*^R132H^* cells, respectively. **F.** 2-HG levels in U87MG-IDH1*^R132H^* cell extracts were approximately 18-fold the levels in U87MG-IDH1*^WT^* or U87MG-mock cells.

To verify the relationship between *IDH1* mutations and 5-ALA fluorescence, we established human U87 MG glioma cells that stably express R132H mutant (IDH1*^R132H^*) or wild type (IDH1*^WT^*) *IDH1*, using lentiviral vectors (Figure 1). We used U87MG cells transduced only with vector as controls (U87MG-mock). We also confirmed that U87MG-IDH1*^R132H^* cells produce 2-HG at significantly higher levels than U87MG-IDH1*^WT^* cells (Figure 1).

**Figure 2:**
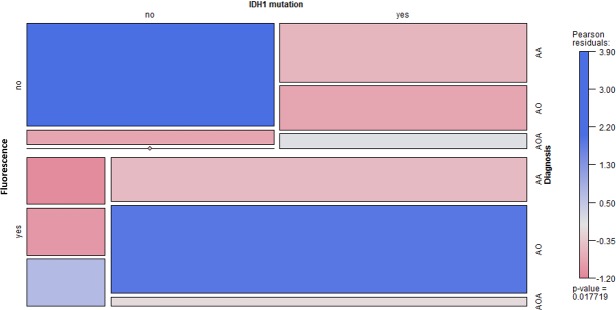
Mosaic plot for 5-ALA fluorescence, *IDH1* mutations, and histological diagnosis (*n* = 35) The colored cells show the magnitude of the Pearson residuals obtained from an independence model. (AA, anaplastic astrocytoma; AO, anaplastic oligodendroglioma; AOA, anaplastic oligoastrocytoma).

**Figure 3:**
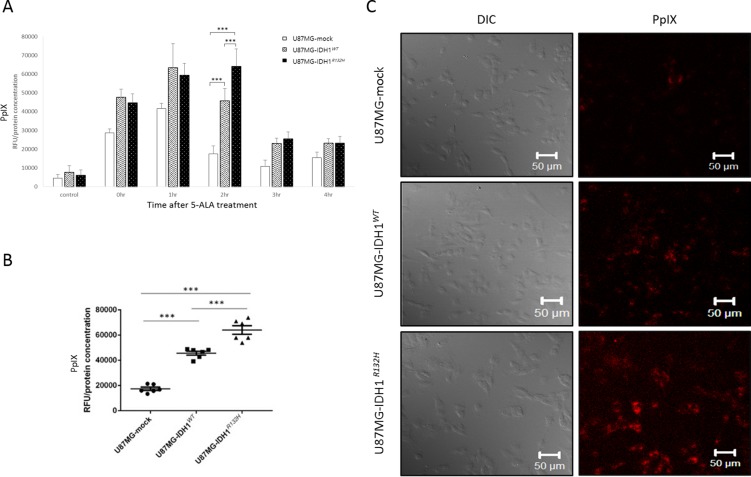
*In vitro* evidence for an association between *IDH1* mutations and 5-ALA fluorescence **A.** PpIX concentrations after 5-ALA treatment, expressed as relative fluorescence units (RFUs) normalized against total cell protein levels. **B.** Accumulation of intracellular PpIX 2 hours after 5-ALA treatment. **C.** Confocal laser scanning microscope images taken 2 hours after 5-ALA treatment. PpIX is visible as red fluorescence, located mainly in the cytoplasm. (λ_ex_ = 405nm, λ_ex_ > 560nm).

From our clinical experience with 5-ALA fluorescence-guided brain tumor surgery, we hypothesized that the difference in fluorescence between malignant glioma and normal brain tissue is a temporary event best observed between 3 and 9 hours after 5-ALA administration [1]. Thus, we performed an *in vitro* time-course analysis measuring 5-ALA-derived PpIX concentrations in a glioma cell line after exposure to 5-ALA. After an 1-hour incubation with 5-ALA, the fluorescence intensity of intracellular PpIX was measured at various time points between 0 to 4 hours with a fluorescence plate reader and normalized to total cell protein concentrations measured using the bicinchoninic acid (BCA) protein assay. Intracellular PpIX fluorescence increased rapidly up to 1 hour after incubation, then diminished in U87MG-IDH1*^WT^* cells (Figure 3A), whereas there was a delay in 5-ALA metabolism in U87MG-IDH1*^R132H^* cells; PpIX fluorescence peaked at 2 hours after incubation, at which time there was a significant difference in PpIX concentration between U87MG-IDH1*^R132H^* and U87MG-IDH1*^WT^* cells (Figure 3B). This difference was associated with differences in fluorescence visualization as well (Figure 3C). These results indicate that the *IDH1* mutation is associated with a lag in 5-ALA metabolism, resulting in temporary differences in the fluorescence activity of malignant glioma cells.

### Citrate, α-KG, and 2-HG levels are selectively altered in IDH1 mutant malignant glioma cells upon exposure to 5-ALA

**Figure 4:**
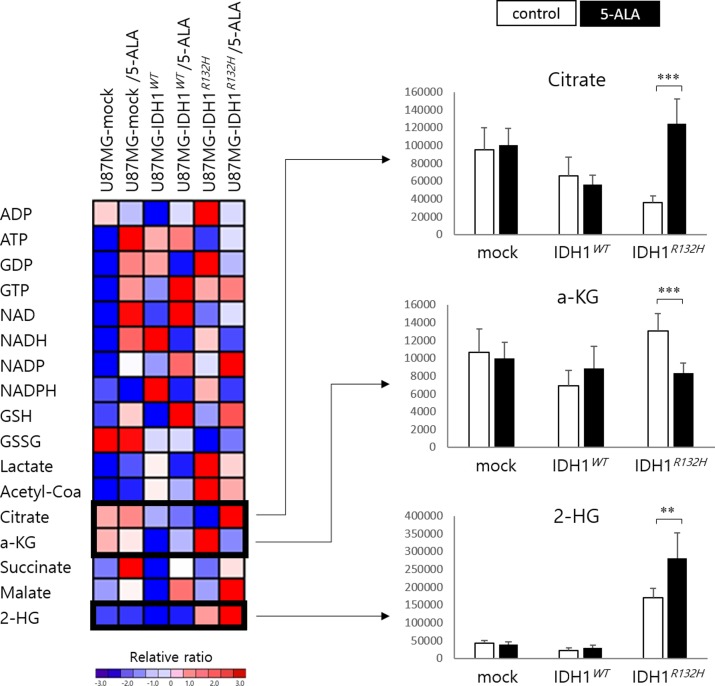
Heat map illustrating the relative ratio of the metabolites expressed in U87MG cells with vector only, the *IDH1* wild type construct, and the *IDH1* mutant construct Metabolites whose levels were significantly different in *IDH1* mutant cells but not wild type cells after 5-ALA treatment are shown in separate histograms.

To screen for metabolic changes that could explain the difference in 5-ALA metabolism between U87MG-IDH1*^R132H^* and U87MG-IDH1*^WT^* cells, we profiled tricarboxylic acid (TCA) cycle intermediates in the lysates of both cell lines, with or without 5-ALA exposure, using liquid chromatography–mass spectrometry (LC-MS). A total of 17 multiple reaction monitorings (MRMs) were performed, and values of detected metabolites were normalized to total protein levels in the cell, as measured by the BCA method. To select candidate metabolites related to enhanced 5-ALA fluorescence in *IDH1* mutant malignant glioma cells, we identified three metabolites (citrate, ɑ-KG, and 2-HG) of which the levels were significantly altered in U87MG-IDH1*^R132H^* cells after 5-ALA treatment (Figure 4). After 5-ALA treatment, citrate increased (3.5 fold; *p* < 0.001) and ɑ-KG decreased (0.6 fold; *p* < 0.001) in U87MG-IDH1*^R132H^* cells but not in U87MG-IDH1*^WT^* cells. Non-treated *IDH1* mutant glioma cells produce an excess of 2-HG relative to wild type glioma cells (7.8 fold; *p* < 0.001); 2-HG levels increased further after 5-ALA treatment (1.7 fold; *p* < 0.005). Levels of other observed TCA intermediates were similar in U87MG-IDH1*^WT^* and U87MG-IDH1*^R132H^* cells.

### Hypothesis for the mechanism of enhanced 5-ALA fluorescence in IDH1 mutant malignant glioma cells

**Figure 5:**
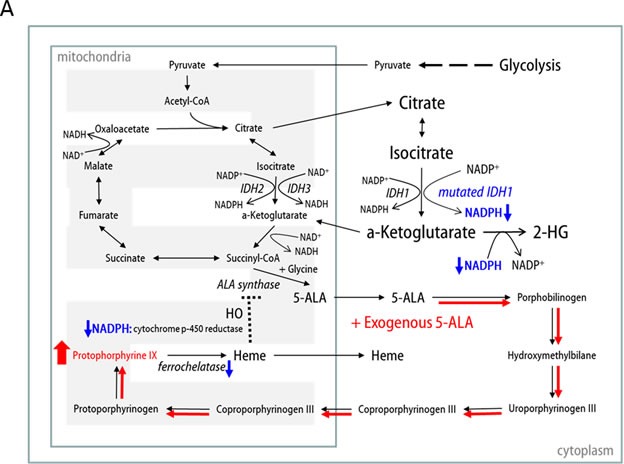

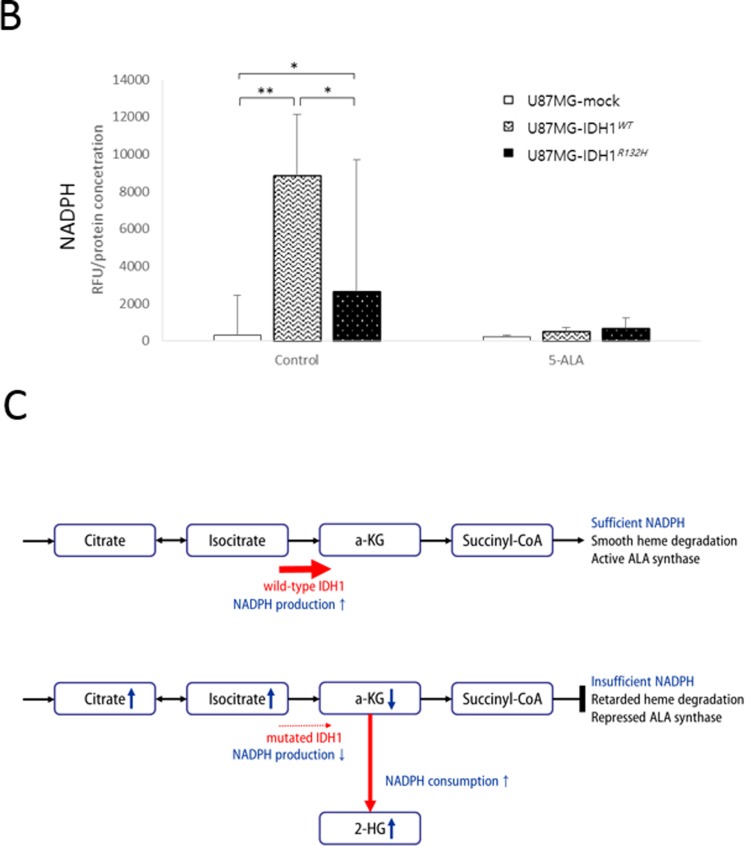
**A.** Schematic of our hypothesis that the lag in 5-ALA metabolism in *IDH1* mutant cells is due to insufficient NADPH. **B**. Intracellular NADPH levels measured by LC-MS. C. Schematic illustration of the metabolic effects of 5-ALA treatment in IDH1*^WT^* and IDH1*^R132H^* cells.

To develop a hypothesis regarding the mechanism of enhanced 5-ALA fluorescence in *IDH1* mutant malignant glioma cells, we reviewed crosstalk between the TCA cycle and the heme synthesis pathway (Figure 5A). Succinyl-CoA from the TCA cycle and glycine are combined by ALA synthase to form 5-ALA [19-23]. ALA synthase is also believed to be the rate-limiting synthetic enzyme in the heme synthesis pathway and is negatively regulated by heme, which is usually degraded by heme oxygenase (HO), via a feedback mechanism [19]. In this step, HO degrades heme in concert with NADPH cytochrome P450 reductase, and it has been shown that HO activity can be increased by NADPH or NADH [24]. Interestingly, one production source of NADPH is the conversion of isocitrate to ɑ-KG by IDH1 or IDH2. Moreover, *IDH1/2* mutations not only decrease the production capacity of NADPH, but also increase consumption of NADPH during the aberrant production of 2-HG from ɑ-KG, a NADPH-dependent reduction process [13]. We confirmed that baseline NADPH levels were lower in U87MG-IDH1*^R132H^* cells than U87MG-IDH1*^WT^* cells (0.3 fold; *p* < 0.05) and that NADPH was almost depleted after 5-ALA treatment in both cell lines (Figure 5B). Therefore, we may explain the lag in 5-ALA metabolism in *IDH1* mutant malignant glioma cell lines by invoking alterations in NADPH metabolism (Figure 5A). If exogenous 5-ALA is administered, the heme pathway is normally hyperactivated to digest the excess 5-ALA; heme is degraded unrestrictedly by HO, given sufficient NADPH, with no accumulation of PpIX. However, if *IDH1* is mutated, heme degradation is disturbed as a result of insufficient NADPH for HO activity, resulting in underexpression of ferrochelatase and, in turn, accumulation of PpIX. This hypothesis is supported by our data on alterations in metabolite levels (Figure 4). In U87MG-IDH1*^WT^* cells, no interference exist from the TCA cycle with the heme synthesis pathway, whereas the conversion of isocitrate to ɑ-KG proceeds normally (Figure 5C). However, ineffective conversion of isocitrate to ɑ-KG, the production of 2-HG from ɑ-KG, and retarded metabolism at the junction of the TCA cycle and heme synthesis pathway due to insufficient NADPH in U87MG-IDH1*^R132H^* cells leads to accumulation of citrate and the depletion of ɑ-KG (Figure 5C).

### Validation of metabolic changes in human WHO grade III gliomas

**Figure 6:**
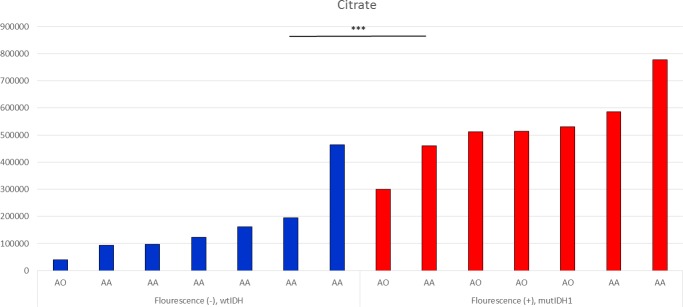
Citrate levels in tumor samples, measured by LC-MS All samples were taken after 5-ALA administration and grouped according to optical evidence of intraoperative fluorescence (AA, anaplastic astrocytoma; AO, anaplastic oligodendroglioma). Significant differences were observed between wtIDH1 without fluorescence group and mutIDH1 with fluorescence group (mean value; 167639.8 *vs* 525871.0, *p* < 0.001).

We created an independent validation set of 14 WHO grade III gliomas treated with 5-ALA fluorescence-guided surgery. The 7 samples were with mutated *IDH1* and positive fluorescence, and the other 7 samples were with wild-type IDH1 and negative fluorescence. We measured metabolites in frozen tumor samples using LC-MS. Among the 13 available MRMs with normalized values, there was a significantly higher level of citrate in fluorescence positive samples than negative ones (Figure 6, *p* < 0.001). However, NADPH, ɑ-KG, and succinyl-CoA were not detected in any of the samples, probably because of excessive consumption due to 5-ALA-induced metabolism.

## DISCUSSION

Fluorescence-guided surgery using 5-ALA is gaining wide use in malignant glioma treatment. [25-28]. Most plausible molecular mechanism involve expression of enzymes, such as ferrochelatase, or transporters such as ABCB6 [27, 28]. However, no study has investigated 5-ALA fluorescence in malignant gliomas from the perspective of metabolomics. The current study documented the relationship between *IDH1* mutations and 5-ALA fluorescence. It is natural to approach the mechanism of 5-ALA fluorescence in malignant gliomas from the standpoint of metabolomics, because the differences of fluorescence between tissues are temporary event indicating differences in metabolism-processing capacity among the tissues. It is a matter of turnover rate of PpIX, rather than accumulation itself, that matters for the mechanism of 5-ALA fluorescence. We propose that NADPH is the key mediator in the varying 5-ALA fluorescence observed between wild type and mutant *IDH1* malignant glioma cells. In the present study, the heme biosynthesis pathway lags in *IDH1* mutant malignant gliomas; 5-ALA overload due to insufficient NADPH can result in a temporary difference in PpIX accumulation relative to wild type *IDH1* malignant glioma cells. It has been shown that mutant IDH1 is responsible for the inhibition of the mutual conversion of isocitrate and ɑ-KG in a NADP+ or NADPH-dependent manner [11, 13, 29, 30]. Recent research implies that reduction of the NADPH production capacity in *IDH1* mutant cells may be a contributing factor in cancer formation [31, 32]. And also, NADPH has been suggested as a promising therapeutic target in glioblastoma cells [33].

*IDH1/2* mutations in glioma are thought to be principal events in early-stage gliomagenesis, and their oncometabolite, 2-HG, is responsible for multiple activities involved in tumor development [34, 35]. In addition, 5-ALA fluorescence is strongly associated with phenotypic tumor aggressiveness in gliomas [5]. Therefore, if there are metabolic changes linking *IDH1* mutations and 5-ALA fluorescence, they may implicate important metabolic steps reflecting malignant transformation in gliomas. One of the key molecules involved in metabolic alterations in cancer is NADPH, which functions as a cofactor in many enzymatic reactions crucial for macromolecular biosynthesis and as a defender against reactive oxygen species (ROS) produced during rapid cancer cell proliferation [36]. There are two sources of NADPH production: the TCA cycle and the pentose phosphate pathway [37]. In the TCA cycle, IDH1/2 and malic enzyme 1 (ME1), of which the levels are altered in many type of cancer cells, contribute to NADPH production [36]. Alternately, overexpression of pyruvate kinase isoform M2 (PKM2) in various type of cancer cells can divert metabolic precursors into the pentose phosphate pathway to produce NADPH [36]. Controlling increased levels of ROS with NADPH is crucial for the survival of cancer cells. Moreover, growing evidences indicate that oncogenesis of IDH1 mutated tumor involves reduced production of NADPH as well as oncometabolite of 2HG production [38]. The role of NADPH in the present study provides insight into its use as a potential target for diagnostic and therapeutic purposes in the future. In that NADPH is more easily detectable than 2-HG by non-invasive diagnostic modalities such as magnetic resonance spectroscopy (MRS), development of NADPH as a surrogate marker for IDH1 mutation and predictive marker of 5-ALA fluorescence can be a possible example of clinical applications.

The mechanism suggested in the present study may not be the only one responsible for 5-ALA fluorescence in gliomas. In fact, 96% of glioblastomas lack *IDH1/2* mutations but show a strong 5-ALA fluorescence [6]. In addition, most low-grade gliomas lack 5-ALA fluorescence and harbor *IDH1/2* mutations [6]. Thus, we used the term “enhanced 5-ALA fluorescence” to describe the result of the hypothesized mechanism, cognizant that there must be another way to explain 5-ALA fluorescence in gliomas as a whole, or at least in glioblastomas. However, in light of the growing evidence of aberrant metabolism involving the TCA cycle in gliomas, we believe that crosstalk between the TCA cycle and the heme pathway is the core mechanism for selective 5-ALA fluorescence. There may be diverse ways to trigger this mechanism that have yet to be discovered. Genomic screening in relation to 5-ALA fluorescence will give insight to this question. The other issue to be validated in further study is the correlation of 5-ALA fluorescence and WHO grade III gliomas with diverse molecular and genetic characteristics. Compared to glioblastomas, both clinical and translational studies on 5-ALA fluorescence focusing on WHO grade III gliomas are rare.

In conclusion, enhanced 5-ALA fluorescence is associated with *IDH1* mutations in malignant gliomas. Reduced production of NADPH plays a role in the lag of the heme synthesis pathway as observed in mutant *IDH1* cells, resulting in the accumulation of intracellular PpIX and enhanced fluorescence. These discoveries provide new insights into cancer metabolism in malignant gliomas and are expected to contribute to glioma management and research. By using a non-invasive method to detect metabolic characteristics specific to *IDH1* mutants, we may be able to select appropriate candidates for fluorescence-guided surgery. NADPH itself can be developed not only as a biomarker, but as a therapeutic target in *IDH1* mutant gliomas. Further investigations into this aberrant metabolic axis in gliomas will broaden our knowledge of gliomagenesis and the mechanism of malignant transformation.

## MATERIALS AND METHODS

### Acquisition of patient and sample data

We used a cohort of 49 patients with histologically-proven WHO grade III gliomas to acquire clinical data and samples, based on a program approved by the Institutional Review Board. For fluorescence-guided surgery, 20 mg/kg of 5-ALA (Gliolan^®^; Medac, Wedel, Germany) was administered orally 3–4 hours before the induction of anesthesia. The main procedure was carried out at 5–7 hours after 5-ALA intake. 5-ALA positive fluorescence was confirmed if there were any red fluorescent spots in the tumor under blue-violet light. The fluorescence status of each area was documented as fluorescence positive or negative by the operating neurosurgeon. The tumor was defined fluorescence positive if at least a focal area was identified as positive fluorescence. Positive areas were collected separately for histological diagnosis and study. All tumor samples used in this study were snap-frozen in liquid nitrogen as soon as possible during the surgery and stored at −80°C. Fluorescence-guided surgeries were done with a Leica M720 OH5 microscope (Leica, Wetzlar, Germany) equipped with an FL400 Fluorescence module or a Zeiss Pentero equipped with a fluorescent 400 nm UV light and filters (Zeiss, Oberkochen, Germany).

Histological diagnosis was performed using the criteria described in the 2007 WHO Classification of Tumours of the Central Nervous System [39]. *IDH1* mutations in patient samples were detected via targeted sequencing, as described previously [40].

### Construction and establishment of wild type and mutant IDH1 cell lines

Human *IDH1* (GenBank accession number NM_005896) cDNA was obtained from Origene (Rockville, MD, U.S.A), and amplified using the polymerase chain reaction. To generate the *IDH1^R132H^* construct, the G395A base pair change was introduced in the *IDH1* open reading frame. Amplified wild type and mutant *IDH1* constructs were subcloned into lentiviral vector CD526A-1 (System Biosciences, Mountain View, CA, U.S.A) via a proprietary Eco cloning method. This vector contains a green fluorescent protein (GFP)-Puromycin fusion dual marker under a Rous sarcoma virus (RSV) promoter, and the cloned insert is expressed under an enhanced, constitutive cytomegalovirus (CMV) promoter (Figure 1A). The primers designed for *IDH1* cDNA cloning were *IDH1*-F, 5′-TCCAGCCTCCGGACTCTAGA-3′, and *IDH1*-R, 5′-AGCAGATACTGGCTTAACTA-3′. The sequence of the cloned cDNA was confirmed by direct DNA sequencing. A control sequence was cloned in the same vector. The control vector could produce a mock virus (not expressing any target, but with the same marker expressed), which was used for negative controls. The recombinant lentivirus was produced by GenTarget Inc (http://www.gentarget.com/, San Diego, CA, USA). Expression lentiviral particles were produced in 293T cells in DMEM medium, collected, and filtered through a 0.45 μm membrane filter (Millipore, Billerica, MA, U.S.A.) before being immediately stored at −70°C. Virus titers were measured via GFP-positive cell counting after transduction into TH1080 cells; the concentration titers were 1.21×10^7^ IFU/mL (mock), 1.09×10^7^ IFU/mL (IDH1*^WT^*), and 1.17×10^7^ IFU/mL (IDH1*^R132H^*).

Human U87MG cells were purchased from the American Type Culture Collection (ATCC, Rockville, MD, USA) and maintained in Roswell Park Memorial Institute 1640 medium (RPMI; WELGENE) containing 10% fetal bovine serum (FBS; GIBCO) in a humidified incubator at 37°C and 5% CO_2_. To express IDH1*^WT^* or IDH1*^R132H^* in cells, U87MG cells were transduced with the corresponding lentivirus for 24 hours in the presence of 4–8 μg/mL polybrene. Transduced cells were selected using puromycin (Cat. No. A7793, Sigma-Aldrich, USA), and transduction efficacy was analyzed via fluorescence microscopy using green filters (Leica, Wetzlar, Germany) and by fluorescence activated cell sorting (FACS) analysis (Figure 1B and 1C). The transduction efficiency of U87MG cells expressing GFP abundantly was over 90% using the lentiviral vector. GFP fluorescence in transduced cells was analyzed using a FACS Calibur cell sorter (BD Bioscience, San Jose, CA, USA) equipped with a 530-nm filter (bandwidth, 15nm), a 585-nm filter (bandwidth, 21 nm), and Cell-Quest software (BD Bioscience). Sorted cells were used for further studies.

Direct sequencing of DNA from each cell line showed the expected sequences for *IDH1^WT^* and *IDH1^R132H^* (Figure 1D). We confirmed the presence of IDH1*^WT^* or IDH1*^R132H^* expression via Western blot (Figure 1E). Cells were lysed in ice-cold lysis buffer [20 mM Tris-HCl (pH 7.5), 150 mM NaCl, 1 mM Na_2_EDTA, 1 mM EGTA, 1% Triton, 2.5 mM sodium pyrophosphate, a1 mM β-glycerophosphate, 1 mM Na_3_VO_4_, 1μg/mL leupeptin, and protease inhibitor cocktail (Sigma)], and the concentration of lysate protein was evaluated using the BCA method (Cat. No. 23227, Pierce™ BCA Protein Assay Kit, Thermo scientific, Rockford, IL, USA). Approximately 30 μg protein was loaded in each lane of a polyacrylamide denaturing gel for electrophoresis. After electrophoresis, protein was transferred to nitrocellulose membranes for blotting. We used a rat monoclonal anti-IDH1 antibody (Cat No. DIA-W09, Dianova, Hamburg, Germany), a mouse monoclonal anti-IDH1 R132H antibody (Cat No. DIA-H09, Dianova, Hamburg, Germany), and a rabbit polyclonal antibody to β-actin (Abcam, Cambridge, England). Primary antibodies were detected using horseradish peroxidase-conjugated antibodies (Santa Cruz Biotechnology, CA, U.S.A.).

Since aberrant overproduction of 2-HG is a hallmark of malignant gliomas with *IDH1* mutations [13], we used LC-MS to confirm elevated 2-HG levels in U87MG-IDH1*^R132H^* cell lines vs. U87MG-IDH1*^WT^* or U87MG-mock cell lines (Figure 1F).

### *In vitro* 5-ALA treatment

We purchased 5-ALA from Sigma-Aldrich (Cat. No. A7793, USA) and dissolved it in phosphate buffered saline (PBS, pH 7.4) to prepare a 500 mM stock solution. It was sterilized by filtration using a 0.2 um pore filter, and wrapped in aluminum foil to avoid light exposure and stored at −20°C. Next, 5×10^4^ cells were seeded into 24-well plates and incubated in 500 μl media overnight. Cells were incubated with 5-ALA (final concentration 1 mM) for 1 hour, rinsed twice with PBS, then incubated in complete medium until analysis. Special care was taken to avoid exposure to light during the experiments.

### Quantification of PpIX fluorescence

To quantify intracellular PpIX fluorescence, we followed a previously described method with modifications [41]. Specifically, after 5-ALA treatment, cells were lysed with 60 μl 0.2% (vol/vol) Triton X-100 on ice for 5 minutes and centrifuged at 13,000 rpm for 5 minutes at 4°C. Next, 5 μl cell supernatant was transferred for the BCA Protein Assay to normalize detected relative fluorescence units (RFUs) of PpIX. To detect PpIX fluorescence, 100 μl methanolic perchloric acid, consisting of 5.6% (vol/vol) perchloric acid in 50% (vol/vol) methanol, was added to the sample and incubated at 37°C in darkness for 15 minutes, then centrifuged at 13,000 rpm for 5 minutes at 4°C. Then, 100 μl supernatant was transferred into 96-well black plates, and fluorescence intensity was measured using the Infinite M200 PRO (TECAN, Switzerland) and Magellan^TM^ software at a fluorescence excitation wavelength of 400±9 nm and an emission wavelength of 645±20nm.

To visualize PpIX fluorescence, microscope coverslips (12 mmФ, Cat No. 01 115 20, Labnet, Germany) were coated with 0.1% gelatin in a 37°C incubator for 1 hour and washed with PBS. Next, 2×10^4^ cells were plated on the coverslips in 24-well plates. A 5-ALA solution (final concentration 1 mM) was added, The 24-well plates were wrapped in aluminum foil to avoid light exposure for 1 hour, and were then rinsed with PBS. After incubation for 2 hours, cells on the coverslip were washed with PBS twice, fixed with 4% paraformaldehyde in PBS at room temperature for 10 minutes, washed again with PBS twice, and mounted using Slow Fade Gold (Cat No. S36936, Invitrogen, USA). Cells were analyzed under 400-fold magnification with a confocal laser scanning microscope (LSM 510 META, Carl Zeiss, Germany). PpIX fluorescence was excited at 405 nm, and images were collected in the red channel using a 560 nm long-pass filter.

### Metabolite screening via LC-MS

Thawed samples were centrifuged at 21,000 g for 20 minutes to remove insoluble materials, then supernatants were transferred into glass vials with micro-inserts for small volume injections. Analysis was carried out using an Agilent 1100 Series liquid chromatography system equipped with a binary LC pump, a degasser, and an auto-sampler set at 4°C (Agilent, CO, USA). Chromatographic separation was performed by injecting 2 μl sample on a ZIC-pHILIC Polymeric Beads Peek column (150×2.1 mm, 5 μm, Merck, Germany) at 35°C and a 0.15 mL/min flow rate, using 10 mM ammonium carbonate (pH = 9.0) in distilled water as mobile phase A and acetonitrile (ACN) as mobile phase B. The linear gradient was as follows: 80% B at 0 minutes, 35% B at 10 minutes, 5% B at 12 minutes, 5% B at 25 minutes, 80% B at 25.1 minutes, and 80% B at 35 minutes.

During the LC-MS experiments, mass spectra were acquired in the negative ion mode using an API 2000 Mass Spectrometer (AB/SCIEX, Framingham, MA, USA) equipped with an electrospray ionization (ESI) source. Parameters of the ESI source operation were as follows: −4.5 kV of ion spray voltage; curtain gas (nitrogen), ion source gas 1 (nitrogen), and ion source gas 2 (nitrogen) pressures at 30, 40, and 80, respectively; temperature of the heater (turbo) gas at 350°C.

MRMs were performed and controlled with Analyst 1.6 Software. Chromatographic peaks were identified by comparing retention times, m/z values and MS2 fragmentation patterns with those in the HMDB (www.hmdb.ca), METLIN (metlin.scripps.edu) and Massbank (www.massbank.jp) databases and those of standards.

### Statistical analyses

Significance of deviance was indicated by Pearson residuals ≥2 or ≤-2, calculated from a chi-square test and expressed by a mosaic plot. All statistical analyses were performed using the free statistical software package R (version 3.0.2;http://www.r-project.org/), and IBM SPSS statistics software (version 21; SPSS Inc). Statistical significance was accepted at a level of *p* < 0.05 (*), *p* < 0.01 (**) or *p* < 0.001 (***). Error bars in histograms represent 95% confidence intervals. Quantitative heat maps visualizing the relative expression of metabolites were produced using GenePattern software modules (www.broadinstitute.org/cancer/software/genepattern; ComparativeMarkerSelection version 10, HierarchicalClustering version 6, and HeatMapViewer version 13) [42].
